# Complete genome sequence of a *Salmonella enterica* strain of serovar Agona associated with an outbreak in France

**DOI:** 10.1128/mra.00867-23

**Published:** 2024-05-29

**Authors:** Karol Romero, Virginie Chesnais, Fabien Vorimore, Mai-Lan Tran, Pierre-Emmanuel Douarre, Jean-Charles Leblanc, Sabrina Cadel-Six

**Affiliations:** 1 Salmonella and Listeria Unit, Laboratory for Food Safety, Anses, Maisons-Alfort, France; 2 SPAAD Bioinformatics Platform, Laboratory for Food Safety, Anses, Maisons-Alfort, France; 3 IdentPath Genomics Platform, Laboratory for Food Safety, Anses, Maisons-Alfort, France; University of Maryland School of Medicine, Baltimore, Maryland, USA

**Keywords:** complete genome, *Salmonella *Agona

## Abstract

We report here the complete genome of one *Salmonella* Agona strain isolated in 2017 from a dried milk powder in France.

## ANNOUNCEMENT

*Salmonella* is a Gram-negative bacterium of the *Enterobacteriaceae* family responsible for foodborne gastroenteritis in humans. Here, we present the complete genome of the *Salmonella enterica* subspecies *enterica* serovar Agona (*S*. Agona) strain 2018LSAL00995, which was received by the Laboratory for Food Safety of Anses for genomic analyses and serotype confirmation during the outbreak in France in 2017 (1).

The whole genome was sequenced using NextSeq (Illumina, Inc., San Diego, CA, USA) and MinION (Nanopore, Oxford Science Park, Oxford, UK) Technologies. Prior to genomic DNA isolation, the strain was cultivated overnight at 37°C in brain heart infusion (BHI) agar. Genomic DNA was extracted separately for Illumina and MinION sequencing from 2 mL of BHI overnight cultures using the Wizard HMW DNA Extraction Kit (Promega, France). The kit was used according to the manufacturer’s instructions for the Illumina sequencing; for the MinION sequencing, DNA was not sheared/vortexed or selected for size. The Illumina library preparation and paired-end sequencing (2 × 150 bp) were performed as previously described by Sévellec et al. (2). MinION library was prepared with 500 ng DNA using the Ligation Sequencing Kit (SQK-LSK109, Oxford Nanopore Technologies, France) according to the manufacturer’s instructions, and long read sequencing was performed using a Flongle (R9.4.1) flow cell on the Oxford Nanopore MinION sequencer for 48 hours.

Default parameters were used for all software unless otherwise specified. Illumina produced 1,988,717 high-quality paired reads with 2 × 150 bp length. The raw fast5 files produced by the MinION were basecalled using Guppy version 6.1.1 using the dna_r9.4.1_450bps_sup config file. The long-read sequencing produced 352,860,769 bases in 242,919 reads. The quality of the MinION read was determined with NanoPlot v1.40.2. The mean read length was 1,452 bp, and the mean quality was 11.4 with read length *N*_50_ of 4,533 bp. About 72.1% of the reads had a quality superior to 10. Reads were then filtered with NanoFilt version 2.8.0 (3) to keep reads with higher quality and length. Quality control of short reads was performed using ConFindr version 0.7.4 (4) to control the absence of intraspecies contamination. For both short and long reads, Kraken2 version 2.1.2 (5) was used to assess the absence of interspecies contamination.

A hybrid assembly was performed using the Unicycler assembler version 0.4.8 (6) combining the sequence data sets generated by both Illumina and Oxford Nanopore Technologies sequencing. Unicycler default parameters were used to obtain a 4,891,541 bp chromosome and a 5,284 bp extrachromosomal element designated p2018LSAL00995. The GC content of the chromosome and p2018LSAL00995 is 52.1%. The serovar Agona was attributed using both SISTR version 1.1.1 (7) and SeqSero2 version 1.2.1 (8), and the multilocus sequence type was found to be ST 13. The chromosome and plasmid sequences were annotated with the National Center for Biotechnology Information Prokaryotic Genomes Annotation Pipeline (9) at http://www.ncbi.nlm.nih.gov/genome/annotation_prok.

## Data Availability

The sequence and annotation data of the complete genome of *Salmonella enterica* subsp. *enterica* serovar Agona strain 2018LSAL00995 are deposited in the NCBI/DDBJ/ENA/GenBank database with the SRA accession numbers CP129904-CP129905.
